# Glypican-4 in pregnancy and its relation to glucose metabolism, insulin resistance and gestational diabetes mellitus status

**DOI:** 10.1038/s41598-021-03454-x

**Published:** 2021-12-13

**Authors:** Carola Deischinger, Jürgen Harreiter, Karoline Leitner, Luna Wattar, Sabina Baumgartner-Parzer, Alexandra Kautzky-Willer

**Affiliations:** Clinical Division of Endocrinology and Metabolism, Gender Medicine Unit, Department of Internal Medicine III, Medical University of Vienna, Waehringer Guertel 18–20, 1090 Vienna, Austria

**Keywords:** Glycoproteins, Biomarkers, Gestational diabetes

## Abstract

Glypican-4 (GPC-4) is an adipokine that enhances insulin receptor signaling. Plasma concentrations were found to be elevated in patients with prediabetes but reduced in type 2 diabetes mellitus. No study on Glypican-4 in pregnancy and pregnancy-related insulin resistance has been published yet. GPC-4 levels were investigated in 59 overweight women throughout their pregnancy at the Medical University of Vienna. GPC-4 levels, fasting insulin, fasting glucose, estradiol, liver and renal parameters, and markers of bone development were assessed before the < 21st week of gestation (GW), and at GW 35–37. GPC-4 levels increased from < 21 GW (mean = 2.38 pg/ml, SD = 0.68 pg/ml) to GW 35–37 (mean = 2.96 pg/ml, SD = 0.77 pg/ml, p < 0.001). At the same time, GPC-4 levels correlated negatively with estimated glomerular filtration rate (eGFR), serum protein and serum albumin levels and were positively related to creatinine and uric acid levels at GW 35–37. Concerning glucose metabolism, GPC-4 levels were inversely related to ISSI-2, fasting insulin and HOMA-IR, however, not significantly different between women with normal glucose tolerance (NGT) and GDM (p = 0.239). In conclusion, GPC-4 levels rose significantly during pregnancy, correlated negatively with fasting insulin and HOMA-IR but might not be related to gestational diabetes mellitus status.

## Introduction

The adipokine glypican-4 (GPC-4) is a cell surface proteoglycan that has been associated with increasing obesity, diabetes mellitus, metabolic syndrome, bone development and non-alcoholic fatty liver disease (NAFLD) in previous studies^1–5^. Adipose tissue acts as an endocrine organ by secreting adipokines, a process which can be dysregulated by obesity^6^. Adipocyte differentiation is promoted by GPC-4, which also improves insulin receptor signaling in the liver and skeletal muscle cells^7,8^. GPC-4 is an independent marker of insulin resistance with levels twice as high in insulin-resistant obese human subjects compared to their BMI-matched insulin-sensitive controls^8^. Unlike other insulin sensitizers, glypican-4 acts directly on the insulin receptor^8^. GPC-4 impacts the key transcriptional factors for adipocyte differentiation (CCAAT/enhancer-binding protein-α (CEBPα) and peroxisome proliferator‐activated receptor‐γ (PPARγ) through insulin-mediated CCAAT/enhancer-binding protein-β (CEBPβ) phosphorylation^7,8^. In lean humans, GPC-4 was more highly expressed in subcutaneous tissue than visceral adipose tissue and correlates with the body-mass index (BMI) and waist-to-hip ratio (WHR)^8–10^. Decreasing GPC-4 expression in subcutaneous tissue was associated with increasing BMI and WHR, whereas increasing GPC-4 in visceral adipose tissue correlated positively with BMI and WHR^9^. Furthermore, serum GPC-4 concentrations were related to renal insufficiency, bone development and correlate positively with systolic blood pressure (SBP), alanine aminotransferase (ALT), aspartate aminotransferase (AST), fasting insulin and homeostasis model assessment for insulin resistance (HOMA-IR) score^2,11^. When investigated in the context of type 2 diabetes mellitus, GCP-4 was increased in patients with impaired glucose tolerance but decreased in those with diagnosed type 2 diabetes mellitus^1^. To some extent, increased insulin production and resistance is a part of normal pregnancy as well. Driven by placental hormones, peripheral insulin resistance and central leptin resistance diverts glucose to the placenta and, thus, the fetus^12,13^. If the mother’s body fails to adapt to these changes, gestational diabetes mellitus (GDM) can develop^14^. GDM is a pregnancy-related form of hyperglycemia, which affects about 2–6% of all pregnant women in Europe^15^ (depending on the region, some studies report up to 24 or 28%^16,17^) and is associated with complications for both mother and child during pregnancy, childbirth and postpartum^18–21^. Risks include preeclampsia, cesarean delivery, fetal macrosomia, intrauterine fetal demise, neonatal hypoglycemia and a 3.5 fold increased risk for the mothers to develop type 2 diabetes mellitus in later life^18,19^.

Due to the association of GCP-4 with increased fasting insulin and HOMA-IR, we hypothesized an increase of GPC-4 during pregnancy due to the rising physiological insulin resistance and, potentially, a connection to GDM status. Therefore, we aimed at investigating GPC-4 in the context of pregnancy and the development of GDM, which, to the best of our knowledge, has not been done before.

## Materials and methods

### Study participants and design

59 pregnant, overweight women from a prospective longitudinal study (Ethics Committee of the Medical University of Vienna, EK Nr. 2022/2012) conducted at the Medical University of Vienna between 2010 and 2014 were investigated for the study at hand. All subjects gave written informed consent for participation in the study^22^. Inclusion criteria were a singleton pregnancy, age ≥ 18 years and a BMI ≥ 25 kg/m^2^. Exclusion criteria were: pre-existing diabetes, chronic and/or infectious diseases, significant psychiatric disorders or inability to follow instructions related to the studies due to language difficulties. All study subjects were monitored and treated during their pregnancy following the national guidelines^23^. As a tertiary health care center taking care of higher risk pregnancies, a high number of cases with GDM is represented in our cohort. GDM was assessed according to the IADPSG/WHO 2013 guidelines^24^. We measured hemoglobin A1c (HbA1c), fasting insulin, fasting glucose, triglycerides (TG), cholesterol, estradiol, osteocalcin, creatinine, uric acid, serum protein, serum albumin, aspartate aminotransferase (AST) and alanine aminotransferase (ALT). All samples were analyzed in our ISO15189 certified central laboratory at the General Hospital in Vienna (AKH Wien). Methods are available under the homepage of the institute of laboratory medicine, www.kilm.at. Estimated glomerular filtration rate (eGFR) was calculated according to the MDRD (modification of diet in renal disease) formula (eGFR (ml/min/1.73m^2^) = 186 × creatinine^−1.154^ × age^−0.203^ × 0.742)^25^. The formula Glucose 0 min* Insulin 0 min/405 was used to calculate the HOMA-IR^26^. HOMA-IR has been proven to display good sensitivity and specificity for insulin sensitivity in pregnancy^27^. To assess the beta-cell reserve, ISSI-2 (insulin secretion sensitivity index), the product of the Matsuda Index and the ratio of the area-under-the-insulin curve to the area-under-the-glucose curve, was used^28,29^.Weight was measured to the nearest 0.1 kg on calibrated electronic scales (SECA 877/888) wearing no shoes and light clothes. Waist circumference was measured twice at the midpoint between the lower border of the rib cage and the iliac crest and hip circumference at the widest portion of the buttocks. An average of the two measurements was recorded. Systolic and diastolic blood pressure and heart rate were measured on the left arm with an appropriate-sized cuff with a BOSO medicus device (Bosch + Sohn, Jungingen, Germany). Patients were in resting position for at least two to three minutes before testing. An average of two measurements taken one min apart was recorded.

### Assay

For the serum GPC-4 analysis, a human ELISA kit from Cloud Clone Corp. (CCC, USA) was used (http://www.cloud-clone.com/products/SEA998Hu.html). The detection range of this kit is 31.2–2000 pg/ml with an inter-assay CV of < 12% and an intra-assay CV of < 10%. Glypican-4 was measured at the visit < 21st GW and GW 35–37 to allow the investigation of pregnancy-related changes in Glypican-4 levels.

### Statistical analysis

Descriptive data analysis was performed for all parameters. Continuous variables were summarized by mean ± SD and categorical variables by counts and percentages. Assumption of Gaussian distribution of parameters was decided by visual assessment of histograms and calculation of skewness using Kolmogorov–Smirnov test. Consequently, the non-parametrically distributed parameter GPC-4 was log-transformed. All women with GPC-4 values outside the reference range of the GPC-4 kit and 2 outliers with values over 2 × 1.5 IQR were excluded from the analysis. Baseline characteristics were analysed with a related-samples Wilcoxon rank test to compare the two time points during pregnancy. A paired samples T-test was performed to evaluate whether GPC-4 levels change during pregnancy. A logistic regression was calculated to see whether GPC-4 levels at GW < 21 were predictive of GDM status in pregnancy. Finally, a univariate ANCOVA was used to investigate differences between NGT and GDM at GW 35–37 correcting for baseline GPC-4 levels as well as potential influencing factors for GDM such as BMI, weight gain, maternal age and fasting insulin and glucose. Pearson’s correlation was used to develop a correlation matrix of the difference in GPC levels and difference in blood parameters, which are related to GPC-4 according to the previously published literature. Pairwise deletion was used for cases with missing records. As this is a post hoc analysis, a power analysis was omitted. For the statistical analysis, SPSS 25.0 (SPSS Inc, Chicago, USA) was used. A two-sided p-value < 0.05 was considered statistically significant.

### Ethics approval and consent to participate

The study was conducted in accordance to the Declaration of Helsinki and approved by the local ethics committee (Ethics Committee of the Medical University of Vienna, EK Nr. 2022/2012). All subjects gave written informed consent for participation in the study.

## Results

### Baseline characteristics

Table 1 shows the clinical characteristics and history of gestational diabetes mellitus of the study population at the baseline visit and GW 35–37. 23.8% of the patients reported to have had GDM in a previous pregnancy, 31.7% to have given birth to a child with over 4000 g in weight at birth. BMI, waist circumference, diastolic blood pressure increased from the baseline visit to GW 35–37. Concerning blood work, levels of GPC-4, fasting insulin, HbA1c, triglycerides, estradiol, osteocalcin, GFR and uric acid increased whereas serum creatinine, protein and albumin decreased in pregnancy. 40.7% (N = 24/59) of all women developed GDM over the course of their pregnancy. 3/24 women with GDM received insulin treatment in addition to dietary and lifestyle recommendations.

**Table 1:** Baseline characteristics and metabolic parameters of visit at < 21st GW and GW 35–37.

N = 59	Baseline visit (< 21st GW)	GW 35–37	p
Age (in years)	33 (± 5)	33 (± 5)	0.696
GDM in previous pregnancy (N)	14/59		
Birth weight > 4000 g in previous pregnancy (N)	19/59		
Week of gestation	15 (± 2)	36 (± 1)	
BMI (in kg/m^2^)	34.67 (± 4.42)	37.45 (± 4.76)	** < 0.001**
Weight (in kg)	95.2 (± 14.7)	103.2 (± 15.7)	** < 0.001**
Waist (in cm)	110.4 (± 10.5)	121.6 (± 10.0)	** < 0.001**
Hip (in cm)	119.8 (± 14.1)	124.6 (± 12.5)	0.054
Blood pressure systolic (in mmHg)	117 (± 11)	120 (± 12)	0.523
Glypican-4 (in ng/ml)	2.38 (± 0.68)	2.96 (± 0.77)	** < 0.001**
Fasting insulin (in µIU/mL)	10.8 (± 5.6)	18.4 (± 14.6)	** < 0.001**
Fasting glucose (in mg/dl)	82 (± 7)	80 (± 11)	0.202
HOMA-IR	2.22 (± 1.29)	3.91 (± 4.01)	**0.001**
ISSI-2	305.67 (± 112)	308.58 (± 105)	**0.013**
HbA1c (in %)	5.0 (± 0.4)	5.2 (± 0.4)	**0.036**
TG (in mg/dL)	131 (± 43)	231 (± 65)	** < 0.001**
Estradiol (in pg/mL)	3739 (± 2372)	17,163 (± 7113)	** < 0.001**
Osteocalcin (in ng/ml)	14.6 (± 5.1)	21.5 (± 9.7)	** < 0.001**
eGFR (in mL/min)	139.92 (± 27.23)	148.07 (± 28.09)	**0.046**
Uric acid (in mg/dl)	3.3 (± 0.7)	3.9 (± 0.9)	** < 0.001**
Serum protein (in g/L)	63.95 (± 3.53)	58.91 (± 48)	** < 0.001**
Serum albumin (in g/L)	39.1 (± 2.5)	34.8 (± 2.1)	** < 0.001**
AST (in U/L)	19 (± 5)	18 (± 4)	0.715
ALT (in U/L)	20 (± 17)	15 (± 5)	0.077

Significant values are in bold.

Continuous variables were summarized by mean ± standard deviation (SD) and categorical variables by counts and percentages.

*GDM* gestational diabetes mellitus, *BMI* Body mass index, *HOMA-IR* homeostasis model assessment for insulin resistance, *ISSI-2* insulin secretion sensitivity index, *HbA1c* hemoglobin A1c, *TG* triglycerides, *eGFR* estimated glomerular filtration rate, *AST* aspartate aminotransferase, *ALT* alanine aminotransferase.

### Glypican-4 levels throughout pregnancy

**Figure 1:**
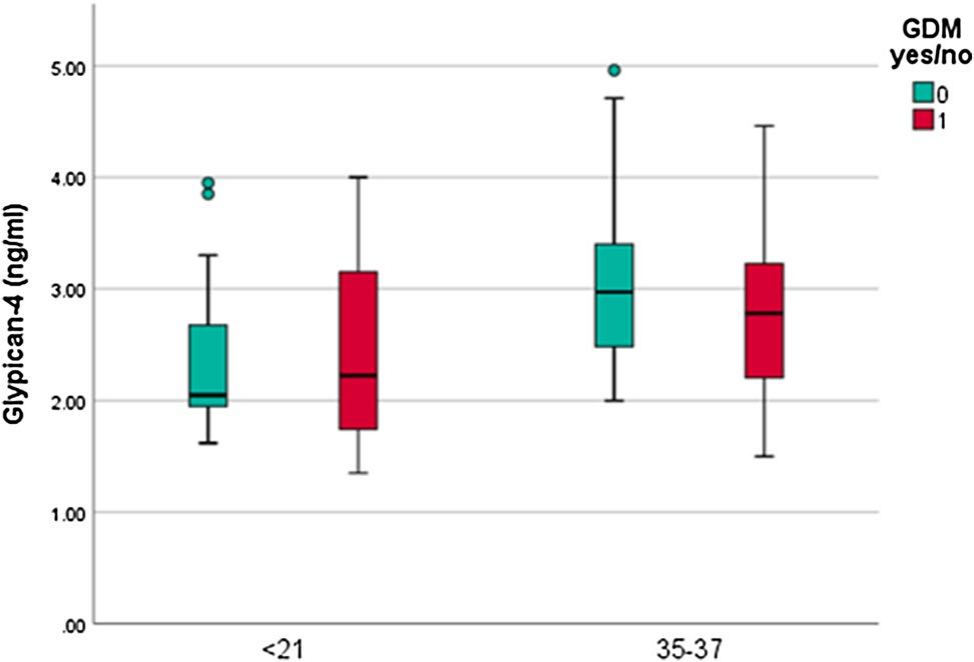
Clustered boxplot of GPC-4 levels by GDM status throughout pregnancy. GPC-4 was significantly higher at gestational week (GW) 35–37 than before GW 21. There was no significant difference in GPC-4 levels between women with NGT and GDM.

As illustrated in Fig. 1, GPC-4 levels increased from < 21 GW (mean = 2.38 pg/ml, SD = 0.68 pg/ml) to GW 35–37 (mean = 2.96 pg/ml, SD = 0.77 pg/ml, p < 0.001, mean difference: − 0.096 ± 0.159).

A logistic regression showed that GPC-4 levels at GW < 21 were not predictive of GDM status in pregnancy (OR = 0.68, p = 0.155). Furthermore, a univariate ANCOVA resulted in no significant differences (corrected mean^NGT^ = 0.475 ± 0.022, mean^GDM^ = 0.429 ± 0.028; p = 0.239) in GPC-4 levels between NGT and GDM at GW 35–37 correcting for baseline GPC-4 levels as well as potential risk factors for GDM such as BMI, weight gain, maternal age and fasting insulin and glucose.

The rise in GPC-4 levels correlated negatively with the change in ISSI-2 during pregnancy (r_p_ = −0.406, p = 0.021). Similarly, as demonstrated in Table 2, GPC-4 at GW 35–37 was negatively associated with fasting insulin (r_p_ = −0.303, p = 0.022) and HOMA IR (r_p_ = −0.306, p = 0.021). Furthermore, GPC-4 levels were positively related to serum creatinine and uric acid and correlated negatively with 1,25-Dihydroxy-Vit D3, eGFR, serum protein and serum albumin. Before GW 21, no association between GPC-4 levels and the investigated parameters could be found.

**Table 2:** Pearson’s correlation analysis of GPC-4 levels during pregnancy at GW < 21 and GW 35–37 as well as the difference in GPC-4 levels (GPC4_diff) correlated with the change in parameters (parameter_diff) from GW < 21 to GW 35–37.

Pearson’ correlation	GW < 21		GW 35–37		GPC-4_diff correlated with parameter_diff	
	r_p_	p	r_p_	p	r_p_	p
Weight gain until GW 35–37		0.047	0.725			
Weight at visit	− 0.145	0.274	0.074	0.583		
BMI at visit	0.098	0.462	0.088	0.511	0.209	0.116
Waist circumference	0.035	0.794	0.085	0.526	− 0.077	0.565
Hip circumference	0.160	0.226	− 0.010	0.938	− 0.060	0.656
Waist-hip-ratio	− 0.145	0.274	0.155	0.246	− 0.048	0.720
Systolic blood pressure	0.115	0.386	0.080	0.550	0.016	0.906
HbA1c	0.211	0.109	− 0.049	0.710	0.083	0.531
Fasting glucose	0.135	0.307	− 0.230	0.080	0.113	0.394
Fasting insulin	− 0.019	0.887	− **0.303**	**0.022**	− 0.041	0.764
HOMA-IR	0.067	0.612	− **0.306**	**0.021**	− 0.045	0.742
ISSI-2	− 0.112	0.411	− 0.261	0.142	− **0.406**	**0.021**
Progesterone	0.068	0.639	0.106	0.427	0.057	0.698
Estradiol	−0.035	0.796	−0.037	0.780	0.057	0.671
Osteocalcin	0.010	0.939	0.215	0.103	0.130	0.328
1,25-Dihydroxy-Vit-D3	− 0.077	0.575	− **0.385**	**0.008**	0.139	0.350
eGFR	− 0.063	0.633	− **0.345**	**0.007**	− 0.034	0.801
Uric acid	− 0.105	0.433	**0.382**	**0.003**	− 0.135	0.314
Serum protein	− 0.001	0.992	− **0.375**	**0.003**	− 0.146	0.274
Serum albumin	− 0.053	0.690	− **0.386**	**0.003**	− 0.028	0.831
ALT	− 0.095	0.473	0.023	0.861	0.08	0.548
AST	0.076	0.572	0.106	0.433	− 0.110	0.420

Significant values are in bold.

*HOMA-IR* homeostasis model assessment for insulin resistance, *ISSI-2* insulin secretion sensitivity index, *HbA1c* hemoglobin A1c, 1,25-Dihydroxy-Vit-D3, *eGFR* estimated glomerular filtration rate, *AST* aspartate aminotransferase, *ALT* alanine aminotransferase.

Significance level is p < 0.05.

## Discussion

To our best knowledge, no evaluation in pregnancy or of women with gestational diabetes mellitus has been done until now. In the present study, GPC-4 increased from < 21 GW to GW 35–37 and was related to fasting insulin, ISSI-2 and HOMA-IR. However, in contrast to studies on patients with type 2 diabetes mellitus, GPC-4 levels were not significantly different between women with NGT and GDM in our analysis. The lack of differences in GPC-4 levels between NGT and GDM was, to some extent, not surprising. Women in both groups were increasingly insulin resistant due to pregnancy-related changes and overweight, both factors by which GPC-4 is significantly influenced^5^.

Increasing GPC-4 levels throughout pregnancy might be explained by the changes in glomerular filtration rate (GFR) and renal plasma flow (RPF) during pregnancy considering the relation of GPC-4 to renal function. GPC-4 was directly related to uric acid, creatinine clearance and correlated negatively with GFR, serum protein and albumin levels in our pregnant cohort. GFR and RPF increase progressively in pregnancy, which leads to elevated sodium loss and higher creatinine clearance^30–32^. Serum albumin, total protein^33^ and creatinine decrease during pregnancy^34^. Uric acid decreases in early pregnancy, followed by a steady incline from mid-pregnancy to term^35^. A Korean study in patients with renal insufficiency corroborates our results^36^. Patient with renal insufficiency showed increased levels of GPC-4 as well as a negative association of GPC-4 levels with GFR and a positive correlation with urinary albumin excretion^36^.

The fact that all participating women were overweight or obese might also be one of the most powerful influencing factors of GPC-4 levels. Except for adiponectin, most adipokines are increased in adiposity and act as mediators of adverse effects associated with obesity^37^. Specifically, glypican-4 was directly related to WHR, the ratio of visceral to subcutaneous fat, fasting insulin, and HOMA-IR score in previous studies^2,5^. We could not corroborate these results as GPC-4 levels were inversely related to fasting insulin, HOMA-IR, ISSI-2 and not associated with BMI or WHR in our analysis. ISSI-2 enables to gauge the beta cell reserve and is higher in pregnant women with NGT than those with GDM^29,38,39^. At this point, we are not able to explain these discrepancies as the exact relationship of GCP-4 with adiposity and glycemic control remains unclear. For instance, GPC-4 has been demonstrated to be increased in patients with impaired glucose tolerance but decreased in those with diagnosed type 2 diabetes mellitus in a previous study^1^.

In most of our GDM cases, dietary changes and physical activity sufficed as treatment, which is in accordance to previously published literature^40^, and only a small fraction (3 patients) required insulin treatment. Potentially, nutritional and/or pharmacological interventions might have restricted maternal weight gain and, thus, affected GPC-4 levels at 35–37 weeks.

As mentioned above, GPC-4 levels were not significantly different between women with NGT and GDM. However, our cohort is homogenous concerning GDM risk factors such as age and BMI, with which GPC-4 is significantly associated^5^. The status of GDM, in contrast to patients with type 2 diabetes mellitus, might, thus, not be the determining factor for differences in GPC-4 levels during gestation.

In conclusion, this study allows a first insight in the potential role of GPC-4 levels in insulin resistance, glucose metabolism and adiposity in pregnancy. A limitation of our study is the fact that we were only able to include overweight women. Further studies including normal-weight women and the addition of pre-pregnancy and postpartum GPC-4 levels would be required to shed light on the role of GPC-4 in pregnancy and insulin resistance.

## Supplementary Information

Supplementary Table S1.

## Data Availability

The datasets used and/or analyzed during the current study are available from the corresponding author on reasonable request.
