# Supplementary material for: Glypican-4 in pregnancy and its relation to glucose metabolism, insulin resistance and gestational diabetes mellitus status
Source: Sci Rep. 2021 Dec 13;11:23898. doi: 10.1038/s41598-021-03454-x (PMC8668887; doi:10.1038/s41598-021-03454-x)
Supplement: Supplementary file 1 — Supplementary Table S1. [file 41598_2021_3454_MOESM1_ESM.docx]

**Glypican-4 in pregnancy and its relation to glucose metabolism, insulin resistance and gestational diabetes mellitus status**

Deischinger Carola^1^, Harreiter Jürgen*^1^, Leitner Karoline^1^, Wattar Luna^1^, Baumgartner-Parzer Sabina^1^, Kautzky-Willer Alexandra^1^

|  | **Baseline visit (<21^st^ GW)** | | **GW 35-37** | |
| --- | --- | --- | --- | --- |
|  | **NGT** | **GDM** | **NGT** | **GDM** |
| Age (in years) | 32 (± 5) | 33 (± 5) |  |  |
| GDM in previous pregnancy (N) | 3/35 | 7/24 |  |  |
| Birth weight > 4000 grams in previous pregnancy (N) | 6/35 | 7/24 |  |  |
| Week of gestation | 15 (±2) | 15 (±2) | 36 (±1) | 36 (±1) |
| BMI (in kg/m²) | 34.6 (± 4.1) | 34.8 (± 5.0) | 37.42 (± 4.39) | 37.57 (± 5.37) |
| Weight (in kg) | 95.9 (± 12.7) | 94.3 (± 17.5) | 103.8 (± 14.1) | 102.2 (± 18.1) |
| Waist (in cm) | 111 (± 8) | 109 (± 11) | 121.7 (± 8.4) | 121.5 (± 12.2) |
| Hip (in cm) | 119 (± 15) | 121 (± 13) | 125.1 (± 13.2) | 123.8 (± 11.6) |
| Blood pressure systolic (in mmHg) | 117 (± 10) | 117 (± 13) | 119 (± 11) | 122 (± 14) |
| Glypican-4 (in ng/ml) | 2.34 (± 0.61) | 2.43 (± 0.78) | 3.08 (± 0.74) | 2.78 (± 0.80) |
| Fasting insulin (in µIU/mL) | 8.8 (± 4.1) | 13.6 (± 6.3) | 13.0 (± 5.9) | 26.3 (± 19.5) |
| Fasting glucose (in mg/dl) | 79 (± 5) | 85 (± 9) | 77 (± 11) | 85 (± 11) |
| HOMA-IR | 1.72 (± 0.81) | 2.95 (± 1.52) | 2.51 (± 1.21) | 5.98 (± 5.58) |
| ISSI-2 | 363 (± 97) | 223 (± 73) | 321 (± 109) | 253 (± 73) |
| HbA1c (in %) | 5.0 (± 0.3) | 5.1 (± 0.5) | 5.1 (± 0.3) | 5.3 (± 0.4) |
| TG (in mg/dL) | 128 (± 49) | 137 (± 32) | 228 (± 70) | 236 (± 57) |
| Estradiol (in pg/mL) | 3585 (± 2181) | 3957 (± 2651) | 16195 (± 5089) | 18575 (± 9262) |
| Osteocalcin (in ng/ml) | 14.1 (± 5.4) | 15.4 (± 4.8) | 19.1 (± 8.5) | 25.0 (± 10.4) |
| eGFR (in mL/min) | 140 (± 31) | 140 (± 21) | 148 (± 32) | 147 (± 22) |
| Creatinine (in mg/dL) | 0.56 (± 0.13) | 0.55 (± 0.07) | 0.53 (± 0.13) | 0.52 (± 0.07) |
| Uric acid (in mg/dl) | 3.2 (± 0.8) | 3.5 (± 0.6) | 3.7 (± 1.0) | 4.3 (± 0.6) |
| Serum protein (in g/L) | 64 (± 4) | 64(± 3) | 59 (± 3) | 59 (± 3) |
| Serum albumin (in g/L) | 39 (± 3) | 39 (± 2) | 34.8 (± 2.2) | 34.9 (± 2.2) |
| AST (in U/L) | 19 (± 5) | 18 (± 5) | 19 (± 5) | 18 (± 4) |
| ALT (in U/L) | 22 (± 21) | 18 (± 8) | 16 (± 5) | 14 (± 5) |

^1^ Department of Internal Medicine III, Clinical Division of Endocrinology and Metabolism, Gender Medicine Unit, Medical University of Vienna, Waehringer Guertel 18–20, 1090, Vienna, Austria

Supplementary table 1: Overview of baseline characteristics and laboratory results at each visit for NGT (normal glucose tolerance) and GDM (gestational diabetes mellitus), respectively. Continuous variables were summarized by mean ± standard deviation (SD) and categorical variables by counts and percentages. BMI= Body mass index, HOMA-IR= homeostasis model assessment for insulin resistance, ISSI-2= insulin secretion sensitivity index, HbA1c=hemoglobin A1c, TG=triglycerides, eGFR=estimated glomerular filtration rate AST=aspartate aminotransferase, ALT=alanine aminotransferase.
